# Prognostic Value of Serial Lactate Dehydrogenase Measurements for Determining Early Mortality in ICU Patients: A Retrospective Cohort Study

**DOI:** 10.3390/jcm15124404

**Published:** 2026-06-06

**Authors:** Hasan Göze, Türkay Akbaş

**Affiliations:** 1 Department of Hematology, Basaksehir Cam and Sakura City Hospital, University of Health Sciences, Istanbul 34480, Türkiye; 2 Division of Intensive Care, Department of Internal Medicine, Düzce University Faculty of Medicine, Düzce 34295, Türkiye

**Keywords:** lactate dehydrogenase, intensive care, mortality, biomarker, prognostic indicator, retrospective cohort

## Abstract

**Background:** This study investigated whether lactate dehydrogenase (LDH) levels measured at ICU admission predict early in-hospital mortality among critically ill medical patients in a single-center retrospective cohort study conducted in Turkey. Specifically, we aimed to (i) determine an optimal LDH threshold; (ii) examine the temporal trajectory of discriminatory performance over 72 h; and (iii) assess LDH as an independent predictor beyond established severity scores. **Methods:** In this single-center retrospective cohort study, 681 adults admitted to a medical ICU between January 2015 and January 2025 were analyzed. Serial LDH measurements were obtained at 0, 24, 48, and 72 h after ICU admission. This study was approved by the Institutional Ethics Committee (Decision No.: 2025/99). ROC analysis was performed under a predefined sensitivity constraint (≥0.70), and time-to-event outcomes were examined using Kaplan–Meier methods and Cox proportional hazards regression. Determinants of maximum LDH were assessed using a GLM with Gamma distribution and log link. **Results:** The 28-day mortality rate was 39.1%. ROC analysis identified an optimal 24-h LDH cut-off of approximately 275 U/L (AUC = 0.650; sensitivity = 0.70). Discriminatory performance improved progressively over time (AUC of 0.632 at baseline to 0.690 at 72 h), suggesting that serial measurements may capture evolving prognostic information more effectively than single-time-point measurements. Kaplan–Meier analyses demonstrated a stepwise decline in survival with increasing LDH across all categorization approaches (all log-rank *p* < 0.001). In multivariable Cox models, log-transformed maximum LDH within the first 72 h was the strongest independent predictor of mortality (HR = 2.2–2.6; *p* < 0.001), demonstrating larger effect sizes than APACHE II, SOFA, and age in fully adjusted models. GLM analysis indicated that male sex was associated with approximately 33% lower expected maximum LDH. **Conclusions:** Admission LDH is an independently predictive and readily obtainable prognostic biomarker for early in-hospital mortality in critically ill medical patients. LDH may complement established ICU risk assessment tools, and its integration into clinical workflows as a triage adjunct warrants further evaluation in prospective multicenter studies.

## 1. Introduction

Intensive care units (ICUs) are specialized clinical environments where critically ill patients with multiple organ dysfunctions and life-threatening systemic complications are managed. Accurate early prognostic prediction in ICU patients plays a crucial role in guiding therapeutic decisions, allocating resources efficiently, and facilitating communication with families. This need is especially relevant in populations characterized by advanced age, high burden of comorbidities, and complex clinical presentations [1,2,3].

ICU-related mortality remains a major global health burden. Worldwide, ICU mortality rates range from 10% to over 40% depending on patient mix, institutional setting, and resource availability. In Turkey, retrospective studies report 28-day mortality rates of 40–50% in medical ICU populations, with the first month following admission being particularly resource-intensive, often requiring prolonged mechanical ventilation, vasopressor support, and multidisciplinary intervention. These data underscore the pressing need for tools that can rapidly identify patients at the highest risk of early death to inform clinical prioritization and resource allocation [1,2,3,4].

While traditional scoring systems such as the Acute Physiological and Chronic Health Evaluation (APACHE) II and the Sequential Organ Failure Assessment (SOFA) are widely used, their applicability often requires time, experience, and the integration of multiple physiological variables. A specific clinical gap that remains unaddressed is the absence of a single, low-cost, and rapidly available biomarker capable of augmenting these scores without additional logistical burden. LDH is routinely measured in most ICUs and becomes available within hours of admission, making it a candidate to fill this gap [4,5].

Lactate dehydrogenase (LDH) is a cytosolic enzyme that catalyzes the final step of anaerobic glycolysis. Serum LDH levels increase markedly in response to tissue damage, hypoxia, systemic inflammation, and cell necrosis, reflecting the disruption of cell membrane integrity. Elevated LDH levels have been documented in a wide range of critical illnesses, including sepsis, acute respiratory distress syndrome, malignancies, post-cardiac arrest states, liver failure, and COVID-19, with consistent associations between elevated LDH concentrations and disease severity or mortality risk [5,6,7,8,9].

Given these characteristics, LDH has attracted attention as a potential prognostic biomarker in critical care settings. Many retrospective studies have demonstrated significant correlations between elevated LDH levels and in-hospital mortality [10,11,12]. However, most of these studies have been restricted to disease-specific cohorts (e.g., sepsis or cardiac arrest), and the prognostic utility of LDH in a general, unselected medical ICU population remains insufficiently characterized [8,13]. Furthermore, existing studies have predominantly relied on single-time-point LDH measurements at admission, precluding assessment of the temporal trajectory of this biomarker during the critical early ICU period. Whether serial LDH values over 72 h provide incrementally superior discrimination compared to a single admission value has not been evaluated systematically in large, general medical ICU cohorts.

We hypothesized that serial LDH measurements over the first 72 h would demonstrate progressive improvements in mortality discrimination, and that maximum LDH within this window would serve as an independent predictor of 28-day mortality beyond conventional severity scores. This study aimed to investigate the association between LDH levels at ICU admission and early in-hospital mortality (within 28 days) in a large cohort of patients admitted to a medical ICU. Specifically, we sought to (i) evaluate the diagnostic performance of LDH and determine an optimal threshold value, addressed through ROC analysis with a predefined sensitivity constraint; (ii) examine temporal changes in discriminatory capacity over the first 72 h, addressed through serial ROC comparisons; and (iii) assess whether LDH serves as an independent predictor of mortality beyond conventional severity scores, addressed through multivariable Cox proportional hazards regression.

## 2. Materials and Methods

### 2.1. Study Design and Setting

This study was designed as a single-center retrospective cohort analysis and was conducted at the Department of Internal Medicine, Faculty of Medicine, Duzce University, Turkey. The patient population consisted of individuals admitted to the medical ICU between 1 January 2015 and 1 January 2025. The study protocol was approved by the Institutional Ethics Committee of Duzce University (Approval Date: 7 April 2025; Decision No.: 2025/99). All procedures adhered to the ethical standards outlined in the Declaration of Helsinki. Informed consent was waived due to the retrospective nature of this study. This study was reported in accordance with the Strengthening the Reporting of Observational Studies in Epidemiology (STROBE) checklist for cohort studies.

Structured research question: In critically ill adults admitted to a medical ICU (Population), do serial serum LDH measurements during the first 72 h (Exposure), compared to established severity scores (Comparator), independently predict 28-day in-hospital mortality (Outcome)?

### 2.2. Patient Selection and Inclusion Criteria

All patients aged 18 years or older who were admitted to the medical ICU during the study period were considered for inclusion. Eligibility required complete electronic medical records with routine laboratory parameters at ICU admission. Patients with missing LDH data or incomplete outcome information were excluded. As a sensitivity consideration, patients excluded for missing LDH data likely represent a small minority; the exclusion criterion was applied uniformly, and clinically, missing LDH at admission most often reflects administrative rather than clinical factors. Potential selection bias and its implications for generalizability are further discussed in the Limitations section.

### 2.3. Data Collection and Variables

Data were retrieved from the hospital’s digital health information system and paper-based ICU records. Data extraction was performed by two investigators who independently reviewed all electronic records and reconciled discrepancies by consensus. A standardized electronic database was pre-specified, shared among the research team, and populated simultaneously and independently by team members. Data collection encompassed the full 10-year study period (January 2015 to January 2025). Collected variables included demographics, length of ICU stay, and ICU mortality status. Laboratory data obtained within the first 6 h of ICU admission included LDH, C-reactive protein (CRP), albumin, hemoglobin, creatinine, and arterial blood gas values. The APACHE II score was calculated retrospectively using the worst available physiological and laboratory values within 24 h of ICU admission [14]. Mortality status was confirmed from the ICU patient tracking system and hospital discharge records. All data were anonymized and stored securely, with access restricted to the study team.

### 2.4. Outcomes

The primary outcome was ICU mortality within 28 days of admission. Secondary outcomes included total ICU length of stay and associations between LDH and other clinical or laboratory parameters.

### 2.5. Statistical Analysis

Analyses were performed using SPSS version 29.0 (IBM Corp., Armonk, NY, USA). Continuous variables were tested for normality using the Kolmogorov–Smirnov test and presented as mean ± standard deviation (SD) or median (interquartile range [IQR]), while categorical variables were expressed as counts and percentages. Group comparisons were performed with the independent samples *t*-test or Mann–Whitney U test for continuous data, and the chi-square or Fisher’s exact test for categorical data.

Serum LDH was measured at admission (0 h) and at 24, 48, and 72 h after ICU admission. For analyses involving 24-h LDH, extreme outliers (z-score > 3) and cases with missing 24-h LDH values were excluded, yielding an analytic sample of 618 patients. LDH was operationalized in three distinct ways: (i) a prognostic threshold for 24-h LDH was derived using ROC analysis under a predefined sensitivity constraint (≥0.70); (ii) percentile-based categories were defined using empirical quartiles (220, 288, and 411 U/L) for Kaplan–Meier survival comparisons; and (iii) clinically anchored categories were constructed based on the local laboratory reference range (upper limit of normal: 225 U/L), with further stratification in approximately 200 U/L increments (225–475, 475–675, and ≥675 U/L).

Time-to-event outcomes were analyzed using Kaplan–Meier methods, with survival distributions compared using the log-rank (Mantel–Cox) test as the primary comparison, supplemented by Breslow and Tarone–Ware tests. Discriminative performance for mortality was assessed using ROC curves with paired comparisons of area under the curve (AUC) across time points.

Associations with mortality were examined using Cox proportional hazards regression, with LDH exposure defined as the logarithm of the maximum LDH measured within the first 72 h. Models were adjusted for age and baseline severity indices (APACHE II and SOFA), and the proportional hazards assumption was evaluated. Variable selection was guided by clinical relevance and prior literature. Multicollinearity was assessed using variance inflation factors; SOFA and APACHE II, although correlated with each other, were retained as they capture distinct dimensions of organ dysfunction and chronic health reserve. Given the event-to-variable ratio in the fully adjusted model, overfitting was considered unlikely.

Determinants of LDH burden were evaluated using a generalized linear model (GLM) with Gamma distribution and log link, with maximum LDH as the dependent variable and sex and ICU length of stay as covariates. These covariates were selected based on evidence linking sex hormones to LDH expression and the physiological rationale that prolonged ICU stay reflects ongoing tissue injury or recovery. We acknowledge that treatment-related variables (e.g., vasopressor use, renal replacement therapy, and mechanical ventilation) were not available as structured data fields in the registry and therefore could not be included as covariates; this represents a limitation, which is discussed below. Parameter estimates were obtained using 1000 bootstrap resamples. Separate univariable binary logistic regression analyses were performed to evaluate LDH quartiles and age groups with 28-day mortality. No formal a priori sample size calculation was performed, as this study utilized a consecutive sample of all eligible patients admitted during the 10-year study period. Two-sided *p*-values < 0.05 were considered statistically significant.

## 3. Results

### 3.1. Patient Characteristics

**Figure 1:**
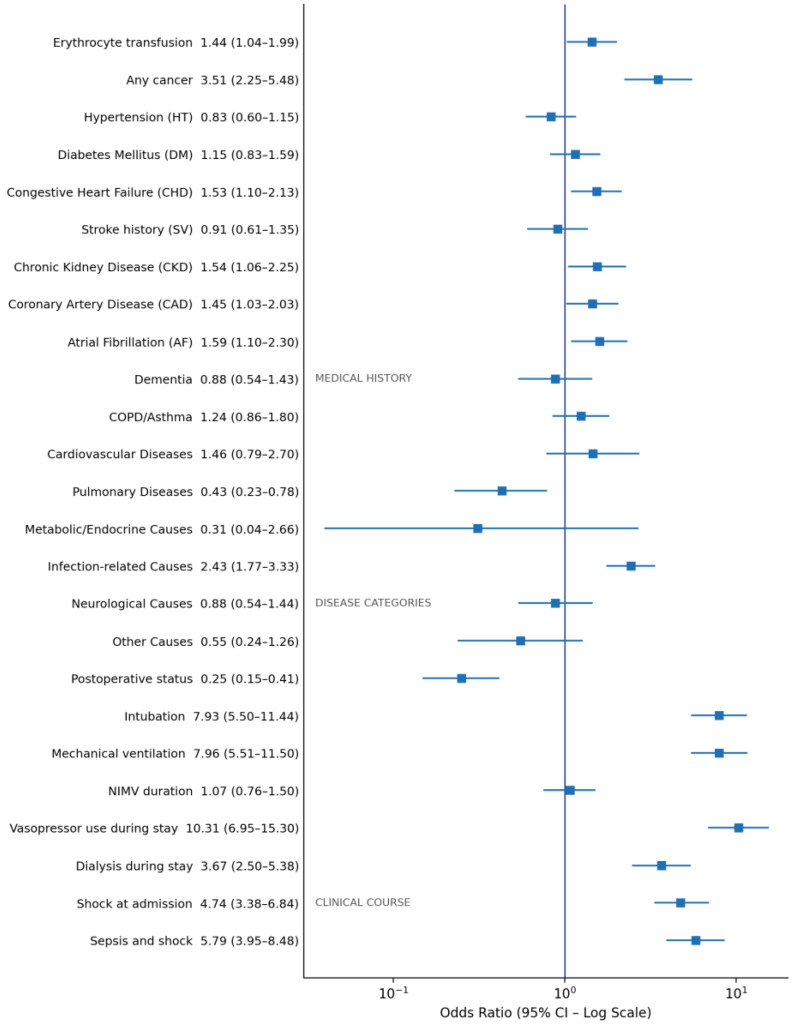
Mortality risk by medical history, disease categories, and clinical course: the forest plot of the study sample (*n* = 681).

A total of 681 patients (mean age = 70 ± 15.5 years; 54.2% male) were analyzed. The 28-day mortality rate was 39.1% (*n* = 266), while 60.9% (*n* = 415) of patients survived at 28 days. The most common admission diagnoses were sepsis or septic shock (34.2%), pneumonia or respiratory failure (22.1%), and acute kidney injury or uremia (10.9%). Other frequent causes included cardiovascular disease (9.0%), hepatic disease (6.6%), neurological disorders (5.7%), malignancy (4.9%), and postoperative or miscellaneous conditions (6.6%). The forest plot in Figure 1 presents a detailed characterization of the study sample with estimates shown alongside 95% confidence intervals.

### 3.2. Univariable Logistic Regression for 28-Day Mortality

Higher LDH (>410 U/L) and age ≥ 73 years were independently associated with increased 28-day mortality in univariable analyses (Table 1). The youngest age group (18–62 years) and the lowest LDH quartile (<220 U/L) were used as reference categories. Notably, LDH in the highest quartile (>410 U/L) was associated with a more than fivefold increase in the odds of 28-day mortality (OR = 5.66, 95% CI = 3.48–9.22; *p* < 0.001), highlighting the strong dose-dependent relationship between LDH elevation and early mortality risk. Odds ratios of >1 indicate higher odds of 28-day mortality when compared with the reference group.

### 3.3. Baseline Characteristics Across LDH Categories

Across 24-h LDH quartiles, baseline severity and laboratory profiles differed significantly for multiple variables, including SOFA score, APACHE II score, renal function (creatinine), oxygenation (PaO_2_/FiO_2_), acid–base parameters (pH, HCO_3_^−^, PCO_2_), lactate, inflammatory markers and cell counts, and serial LDH measurements (all *p* < 0.05). When patients were dichotomized at the ROC-derived 24-h LDH cut-off (~275 U/L), those above the cut-off had significantly higher illness severity (APACHE II, SOFA), lower neurological status (GCS), worse oxygenation (PaO_2_/FiO_2_), impaired renal function (creatinine), higher lactate, and more pronounced inflammatory and tissue injury markers (CRP, AST, ALT, bilirubin), as well as significantly different acid–base parameters (HCO_3_^−^, PCO_2_) and lower nutritional markers (albumin and total protein) (all *p* < 0.05). Age, ICU and hospital length of stay, baseline glucose, pH, PO_2_, mean arterial pressure, hemoglobin, RDW, eosinophils, and basophils did not differ significantly between LDH groups defined by the ROC-derived cut-off (all *p* > 0.05).

### 3.4. ROC Analysis and Optimal LDH Cut-Off Values

**Figure 2:**
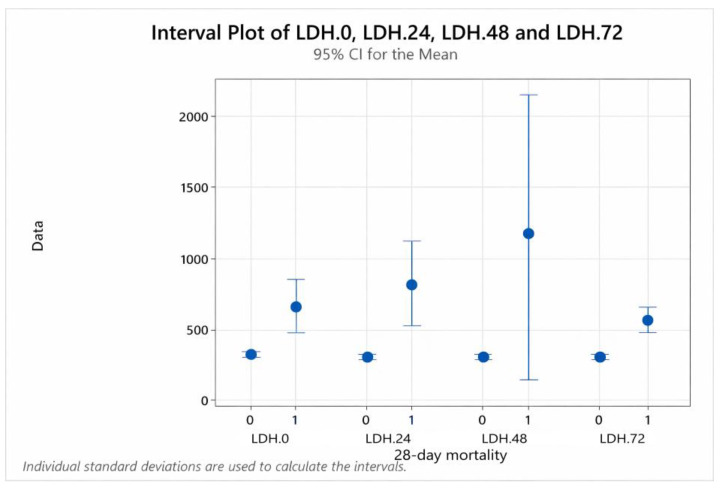
Interval plot of mean LDH values with 95% confidence intervals by mortality status (0/1) and time point.

**Figure 3:**
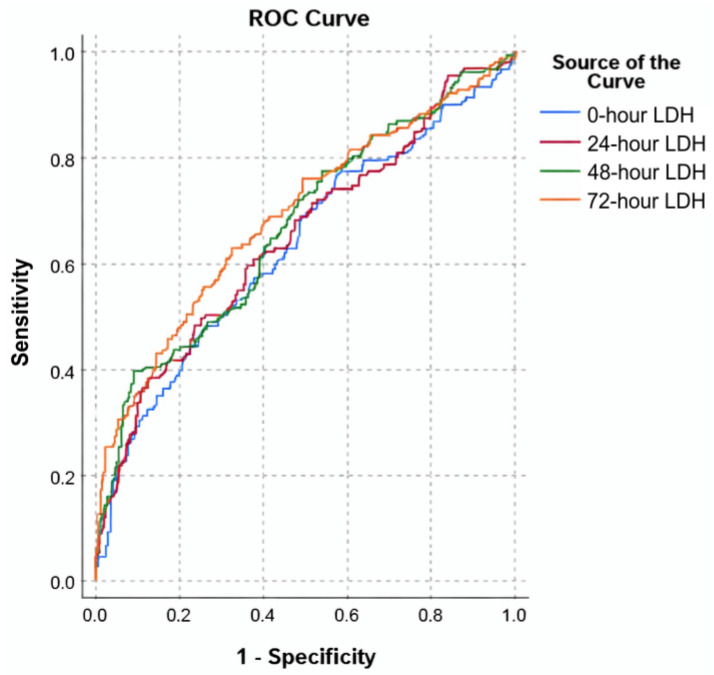
ROC curves for LDH at baseline and at 24, 48, and 72 h after ICU admission.

Across the cohort, LDH levels measured at baseline and at 24, 48, and 72 h were consistently higher among non-survivors than survivors, and LDH increased in parallel with mortality risk. ROC analyses for 28-day mortality demonstrated a progressive improvement in discriminatory performance over time: AUC values were 0.632 at baseline, 0.650 at 24 h, 0.668 at 48 h, and 0.690 at 72 h (Figure 2 and Figure 3).

**Figure 4:**
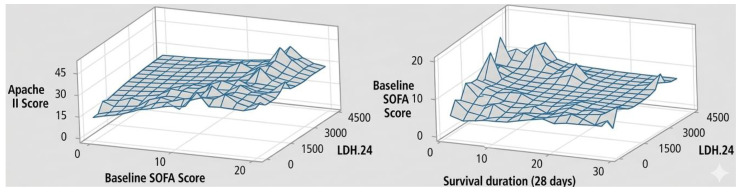
Three-dimensional plots illustrating the relationships between baseline SOFA score, APACHE II score, and 24-h LDH in relation to 28-day mortality outcomes.

As shown in Figure 4, 24-h LDH was weakly but significantly correlated with SOFA (r = 0.224, *p* < 0.001) and APACHE II scores (r = 0.198, *p* < 0.001), while SOFA and APACHE II scores were strongly correlated with each other (r = 0.738, *p* < 0.001).

ROC analyses under the sensitivity ≥ 0.70 constraint were used to identify optimal LDH cut-off values of approximately 292 U/L at baseline (sensitivity = 0.705, specificity = 0.549), 275 U/L at 24 h (sensitivity = ~0.70, specificity = ~0.56), 282 U/L at 48 h (sensitivity = 0.701, specificity = 0.563), and 276 U/L at 72 h (sensitivity = 0.712, specificity = 0.558).

### 3.5. Kaplan–Meier Survival Analyses

Kaplan–Meier survival analyses using predefined 24-h LDH categories showed a clear stepwise decline in survival with increasing LDH levels, with significantly different survival distributions across groups (log-rank, Breslow, and Tarone–Ware tests, all *p* < 0.001). Mean and median survival times decreased monotonically from the lowest to the highest LDH group, demonstrating a dose–response relationship between 24-h LDH and survival.

**Figure 5:**
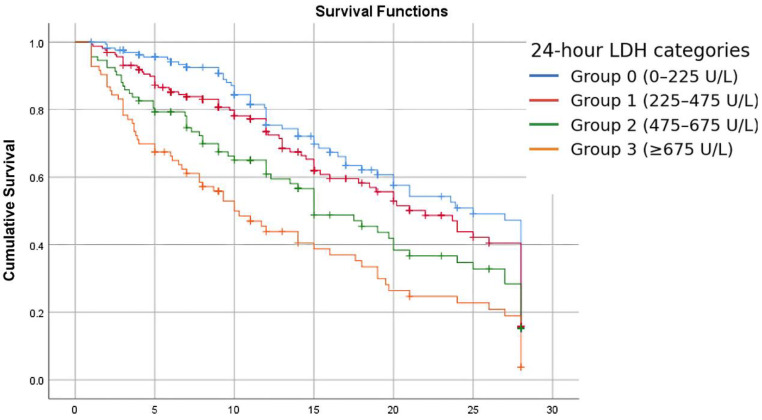
Kaplan–Meier survival curves according to laboratory-anchored 24-h LDH categories.

Using laboratory-anchored categories (0–225, 225–475, 475–675, and ≥675 U/L), median survival decreased stepwise, i.e., 25 days, 22 days, 15 days, and 10 days, respectively, with significantly different survival distributions across groups (all tests *p* < 0.001; Figure 5).

**Figure 6:**
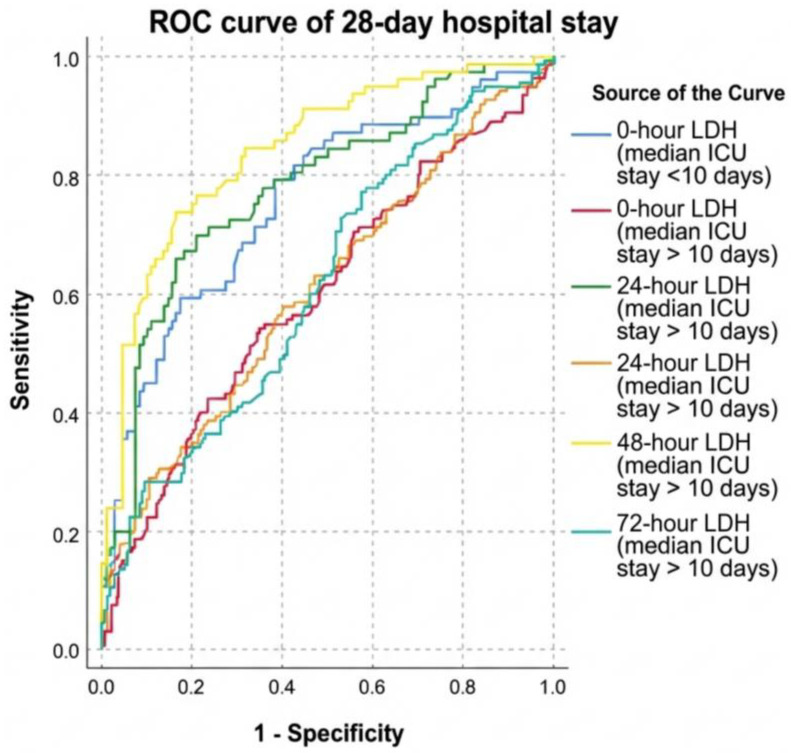
Kaplan–Meier survival curves according to percentile-based 24-h LDH quartiles.

**Figure 7:**
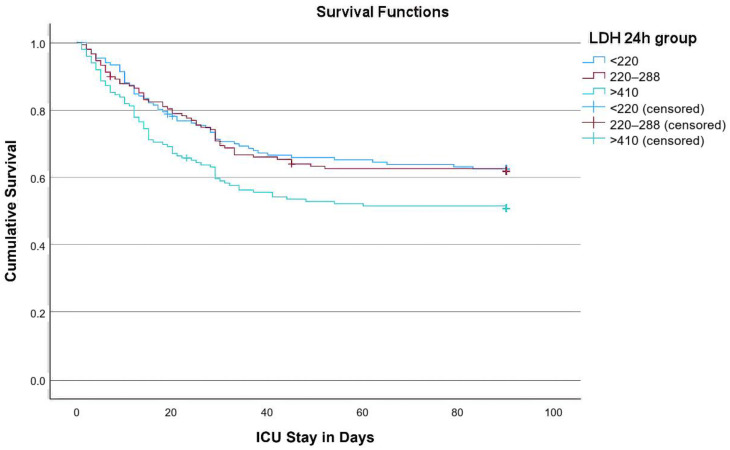
Kaplan–Meier survival curves stratified by percentile-based LDH cut-offs over the 28-day follow-up period.

Using percentile-based grouping (cut-offs: 220, 287.5, and 410 U/L), Kaplan–Meier analysis demonstrated a significant difference in survival across 24-h LDH quartiles (log-rank *p* = 0.041, Breslow *p* = 0.029, Tarone–Ware *p* = 0.034). Mean survival was 64.6 days in the LDH < 220 U/L group, 63.6 days in the 220– 288 U/L group, and 54.7 days in the LDH >410 U/L group. Early mortality was particularly pronounced in the LDH > 410 U/L group, with 75% of patients dying within the first 14 days (Figure 6 and Figure 7).

### 3.6. Cox Proportional Hazards Regression

**Figure 8:**
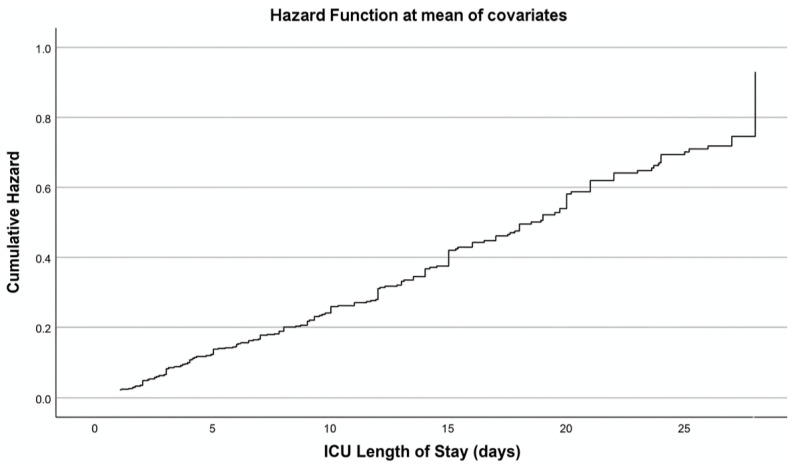
Cumulative hazard plots for log-transformed maximum LDH over the first 28 days of ICU stay.

In Cox proportional hazards models, log-transformed maximum LDH within the first 72 h was consistently the strongest and most stable independent predictor of mortality (HR = 2.2–2.6; *p* < 0.001). The effect size of LDH (HR = 2.2–2.6) was consistently larger than that of APACHE II and SOFA across model specifications, indicating that LDH captured prognostic information not fully represented by composite severity scores. Age did not demonstrate a consistent independent association after adjustment for LDH and severity indices, suggesting that LDH may partially mediate the age–mortality relationship through its reflection of cumulative physiological deterioration. The association between LDH and mortality was consistent across admission service, sex, and sepsis/septic shock status, with no significant effect modification, supporting generalizability within this medical ICU population (Figure 8).

Regarding sex differences in LDH levels, in the GLM analysis, male sex was associated with approximately 33% lower expected maximum LDH compared to females. However, the main effect of sex on mortality was not statistically significant in fully adjusted Cox models, suggesting that the sex–LDH relationship reflects differences in LDH distribution rather than a direct sex effect on the LDH–mortality pathway. This finding may reflect sex-specific differences in muscle mass, metabolic physiology, or underlying diagnosis mix, and warrants further investigation.

### 3.7. Generalized Linear Model: Determinants of Maximum LDH

In the GLM with Gamma distribution and log link, the overall model was statistically significant (likelihood ratio χ^2^ = 116.996, *p* < 0.001). Male sex (reference: female) was associated with lower maximum LDH levels (β = −0.397, 95% CI = −0.543 to −0.251; *p* < 0.001), corresponding to approximately 33% lower expected maximum LDH in males. Longer ICU length of stay was also associated with lower maximum LDH (β = −0.034 per day, 95% CI = −0.041 to −0.026; *p* < 0.001), corresponding to an approximate 3.3% decrease in expected maximum LDH per additional ICU day. Model fit indices indicated adequate fit (deviance/df ≈ 1.02). In partially adjusted models, female sex was associated with higher mortality risk; however, the main effect of sex was not statistically significant in fully adjusted models.

## 4. Discussion

This study demonstrates that serum LDH provides moderate but reproducible discrimination for early in-hospital mortality in a general medical ICU population and remains an independent predictor of death across ROC analysis, survival modeling, and multivariable Cox regression. The AUC values of 0.63–0.69 are consistent with moderate discrimination, which is the expected range for a non-specific tissue injury biomarker in a heterogeneous ICU population. These values should be interpreted in this context rather than as indicators of strong standalone predictive accuracy. Nonetheless, the independent prognostic contribution of LDH, demonstrated across multiple analytical frameworks and confirmed after adjustment for APACHE II and SOFA, supports its value as a complementary rather than competing marker.

The biological plausibility of LDH as a mortality predictor in critical illnesses rests on its role as a marker of cellular energy failure and tissue necrosis. Under conditions of hypoxia and metabolic stress, the shift to anaerobic glycolysis leads to lactate accumulation and LDH release from damaged cells. Systemic inflammation amplifies this process through cytokine-mediated mitochondrial dysfunction and endothelial injury.

**Figure 9:**
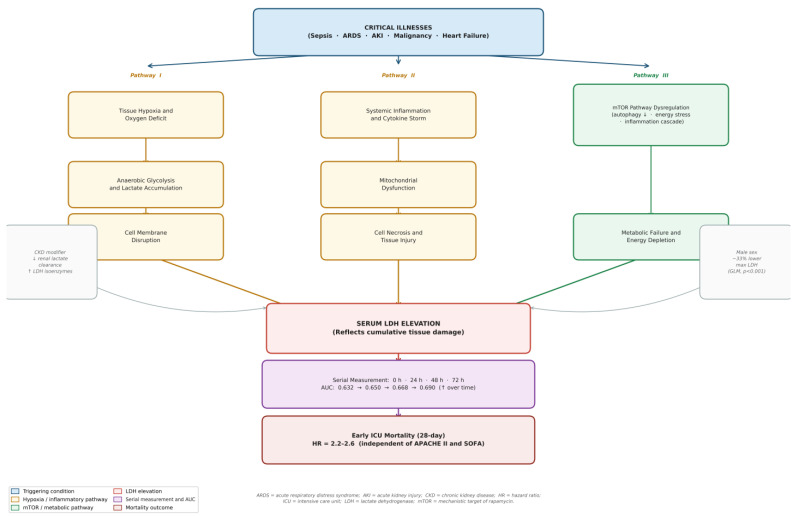
Proposed pathophysiological mechanisms linking serum lactate dehydrogenase (LDH) elevation to multi-organ dysfunction and early mortality in critically ill ICU patients. LDH release reflects cellular energy failure, tissue necrosis, and end-organ damage across hepatic, renal, hematologic, and pulmonary compartments. Abbreviations: LDH, lactate dehydrogenase; ICU, intensive care unit; SOFA, Sequential Organ Failure Assessment; APACHE II, Acute Physiology and Chronic Health Evaluation II; CKD, chronic kidney disease; mTOR, mechanistic target of rapamycin.

Furthermore, the mechanistic target of rapamycin (mTOR) pathway has been increasingly implicated in the metabolic and immune dysregulation that characterizes critical illnesses. As a central integrator of nutrient and stress signals, mTOR governs cellular energy homeostasis by coordinating protein synthesis and suppressing autophagy, and its dysregulation is a recognized feature of diverse metabolic and inflammatory states [15]. In the setting of sepsis and critical illnesses, sustained mTORC1 activation has been shown to inhibit autophagic flux and to promote a glycolytic, pro-inflammatory immune-cell phenotype, thereby contributing to the inflammatory cascade and immune dysfunction associated with adverse outcomes [16,17]. Although a direct causal link between mTOR signaling and circulating LDH has not been established, this glycolytic and pro-inflammatory shift is mechanistically consistent with the metabolic milieu that elevated LDH is thought to reflect, providing a plausible biological framework linking the two. Integrating this mechanistic framework suggests that LDH elevation in ICU patients reflects a confluence of hypoxic tissue injury, inflammatory stress, and metabolic failure, all of which are pathophysiologically linked to poor outcomes (Figure 9).

There is an unmet clinical need for inexpensive, reliable, and rapidly available biomarkers in emergency and critical care settings. Unlike composite severity scores, LDH is a single, routinely measured laboratory value immediately available at the bedside. Our findings indicate that LDH provides a reproducible prognostic signal, demonstrated consistently across multiple analytical approaches, and may complement existing biomarkers in enhancing ICU risk stratification.

Our findings are consistent with the prior ICU literature reporting the moderate discrimination of LDH for mortality prediction (AUC = approximately 0.64–0.67) [18,19]. The optimal 24-h cut-off (275 U/L) was closely aligned with previously reported risk inflection points around 268 U/L [18]. While previous studies confirmed the LDH–mortality association, our study adopts a more comprehensive analytical approach, integrating ROC-derived thresholds, quartile-based stratification, and clinically anchored categories. Furthermore, LDH remained an independent predictor after adjustment for APACHE II and SOFA, supporting its prognostic value beyond conventional indices [10,18,20].

The clinical benefits of serial rather than single-time-point LDH measurements deserve discussion. Serial monitoring allows the detection of LDH trajectories (whether rising, stable, or declining), which may provide more nuanced prognostic information than a single admission value. Practically, LDH is measured at minimal additional cost in most ICUs. Challenges include potential inter-laboratory variability in LDH assay methods and reference ranges, which limit direct threshold comparability across institutions. Additionally, as LDH is non-specific, its prognostic interpretation requires integration with the clinical context. Future studies should evaluate whether LDH trajectory patterns (e.g., persistently elevated vs. early declining) predict outcomes differentially.

Sample characteristics from studies in Turkey appear broadly comparable to our cohort. A recent single-center retrospective study reported a mean age of 64 years and a mortality rate of 50%, broadly consistent with ours [21], although our mean age of 70 years reflects an older population with potentially higher comorbidity burden.

Our ROC analyses confirmed progressive improvement in discriminatory performance over time (AUC of 0.632 at baseline to 0.690 at 72 h), supporting the prognostic advantage of serial over single-time-point LDH measurements [22].

LDH serves as a marker of tissue damage and cellular injury, and its elevation reflects target organ damage across various critical illnesses [20,23]. The dose-dependent relationship between LDH and mortality, with a threshold of around 275 U/L, reinforces its utility for risk stratification.

In partially adjusted models, female sex was associated with higher mortality risk, consistent with studies reporting higher adjusted mortality in women in specific ICU populations such as severe sepsis and cardiac arrest [24]. The diagnosis-dependent nature of sex differences aligns with prior evidence that sex effects on ICU mortality vary substantially with underlying conditions [24]. After full adjustment, the sex–mortality association was not significant, potentially being confounded by clinical severity. The GLM finding that male sex is associated with approximately 33% lower maximum LDH likely reflects biological differences in baseline LDH levels related to muscle mass and sex hormone effects on metabolic pathways, rather than a causal influence on mortality.

Chronic kidney disease (CKD) may modulate LDH through impaired renal lactate clearance, altered LDH isoenzyme expression, and tubular cell injury [25,26,27,28]. In incident hemodialysis patients, LDH > 280 U/L independently predicts mortality [29], and in diabetic kidney disease, higher LDH is associated with increased cardiovascular mortality [30]. In our cohort, discriminatory performance was maintained across CKD strata, supporting LDH’s prognostic utility in this high-risk subgroup.

The observed differences in bicarbonate (HCO3-), PCO2, and albumin between high- and low-LDH groups reflect the association between LDH elevation and tissue hypoperfusion, metabolic acidosis, and nutritional depletion in critically ill patients [31,32,33].

Several limitations should be acknowledged. First, the retrospective single-center design limits generalizability. Second, LDH is non-specific; future studies should explore its additive value alongside diagnosis-specific biomarkers. Third, treatment-related variables (e.g., vasopressors and renal replacement therapy) were unavailable as structured covariates, representing a potential source of residual confounding that we cannot fully address with the available data. Fourth, inter-laboratory variability in LDH assay calibration and reference ranges may affect threshold generalizability across institutions. Fifth, an external validation cohort was not available; prospective multicenter validation is warranted.

### Study Strengths and Contributions

This study makes several contributions to the existing literature. First, by incorporating serial LDH measurements at four time points (0, 24, 48, and 72 h), this study provides empirical evidence for the temporal dynamics of LDH’s prognostic performance, advancing beyond single-time-point analyses that dominate the prior literature. Second, the use of three complementary LDH operationalizations (ROC-derived, quartile-based, and clinically anchored) bridges statistical and bedside-clinical utility more comprehensively than previous reports. Third, the large sample size (*n* = 681) from a 10-year consecutive cohort enhances statistical power and reduces selection bias. Fourth, the consistency of LDH’s prognostic signal across sex, admission diagnosis, and CKD status supports its generalizability within heterogeneous medical ICU populations. Finally, the demonstration that LDH provides independent prognostic information beyond APACHE II and SOFA, two of the most widely used ICU scoring systems, underscores its potential clinical utility as a low-burden adjunct tool.

## 5. Conclusions

This study demonstrates that elevated LDH levels at ICU admission are strongly and independently associated with early in-hospital mortality in a general medical ICU population. LDH integrates multiple pathophysiological mechanisms, including cellular hypoxia, systemic inflammation, metabolic stress, and tissue injury, and captures prognostic information beyond that provided by APACHE II and SOFA scores alone. Given its simplicity, rapid availability, and low cost, LDH may serve as a practical adjunct to established clinical scoring systems for early risk stratification. In practical terms, LDH could be incorporated into admission triage protocols as a first-pass risk flag: patients with LDH > 275 U/L at 24 h may benefit from escalated monitoring, earlier multidisciplinary review, or goals-of-care discussions. Serial measurement over 72 h may additionally support the dynamic reassessment of mortality risk. Prospective multicenter studies are warranted to validate these thresholds and to evaluate whether LDH-guided management strategies can improve ICU outcomes.

## Figures and Tables

**Table 1:** Univariable binary logistic regression analysis of LDH quartiles and age groups for 28-day mortality.

Predictor	Category (vs. Reference)	OR (Exp(B))	95% CI	*p*-Value
LDH quartiles	220–287 vs. <220	0.93	0.56–1.55	0.771
	288–410 vs. <220	1.56	0.96–2.53	0.073
	>410 vs. <220	5.66	3.48–9.22	<0.001
Age groups	63–72 vs. 18–62	1.41	0.91–2.20	0.126
	≥73 vs. 18–62	1.59	1.08–2.32	0.018

## Data Availability

The raw data supporting the conclusions of this article will be made available by the authors on request.

## Abbreviations

APACHE II	Acute Physiology and Chronic Health Evaluation II
AUC	Area Under the Curve
CI	Confidence Interval
CKD	Chronic Kidney Disease
CRP	C-Reactive Protein
GLM	Generalized Linear Model
HR	Hazard Ratio
ICU	Intensive Care Unit
IQR	Interquartile Range
LDH	Lactate Dehydrogenase
OR	Odds Ratio
ROC	Receiver Operating Characteristic
SD	Standard Deviation
SOFA	Sequential Organ Failure Assessment
